# Microbiome Diversity in Seafood Factories via Next-Generation Sequencing for Food Safety Management System (FSMS) Certifications in Malaysia

**DOI:** 10.3390/foods14091517

**Published:** 2025-04-26

**Authors:** Shuping Kuan, Nyuk Ling Chin, Tuan Poy Tee, Noor Zafira Noor Hasnan

**Affiliations:** 1 Department of Process and Food Engineering, Faculty of Engineering, Universiti Putra Malaysia, UPM, Serdang 43400, Selangor, Malaysia; kuanshuping.moh@gmail.com (S.K.); noorzafira@upm.edu.my (N.Z.N.H.); 2 Food Safety and Quality Division, Penang State Health Department, George Town 11600, Penang, Malaysia; 3 Department of Animal Science, Faculty of Agriculture, Universiti Putra Malaysia, UPM, Serdang 43400, Selangor, Malaysia; ttpoy@upm.edu.my

**Keywords:** food safety management system (FSMS), Hazard Analysis Critical Control Point (HACCP), food-contact surfaces, MeSTI, food safety, foodborne

## Abstract

Next-Generation Sequencing (NGS) technology was applied to evaluate Food Safety Management System (FSMS) performance in seafood-processing factories by exploring microbiome diversity alongside traditional methods for detecting foodborne pathogens. A total of 210 environmental swabs collected from processing zones in six factories underwent 16S rRNA amplicon sequencing. FSMS-certified factories exhibited significantly higher species richness, with alpha diversity *p*-values of 0.0036 for observed ASVs, 0.0026 for Faith’s PD and 0.032 for Shannon. Beta diversity analysis also revealed significant differences, with p-values of 0.001 for Bray–Curtis, unweighted UniFrac and Jaccard. Pathogens like *Listeria monocytogenes*, *Salmonella* spp. and *Bacillus cereus* were present in “uncertified” factories but absent in the “certified” factories. The “certified” factories had a significantly higher proportion of lactic acid bacteria (LAB) genera (70.22%) compared to “uncertified” factories (29.78%). The LAB genera included *Streptococcus*, *Lactococcus*, *Lactobacillus* and others. NGS has demonstrated superior capability by providing comprehensive microbiome detection, including the unculturable microorganisms and insights into microbial diversity, so it lacks the limitations that come with traditional culturing. These findings highlight the potential for leveraging beneficial microbes in bioremediation and pathogen control to enhance FSMS effectiveness in seafood-processing environments.

## 1. Introduction

The increase in seafood-associated outbreaks and recalls annually has resulted in more attention being paid to and a greater emphasis being placed on the critical need for robust Food Safety Management System (FSMS) in seafood processing to prevent foodborne diseases [1]. Malaysia, a key player in seafood exports valued at USD 714.1 million [2], relies on robust FSMS implementation, verified through regulatory audits and surveillance to mitigate microbiological risks in seafood-processing environments for both domestic and international markets [3]. However, many Malaysian seafood processors, particularly microenterprises, struggle to achieve FSMS certification due to weak enforcement and a lack of perceived necessity for compliance in domestic markets [4,5]. This non-compliance poses significant public health risks, including antimicrobial-resistant foodborne pathogens that threaten both consumer safety and business integrity [6,7].

Traditional FSMS assessments in food factories predominantly rely on culturable microorganism identification, which sometimes fails to capture the diversity of unculturable microbiomes present on seafood-processing equipment due to tedious culturing procedures and long laboratory turnaround times [8,9]. These limitations can introduce errors and variability, coupled with a lack of sensitivity in detecting low level of pathogens [10]. Recent outbreaks, such as Vibrio-related incidents, have highlighted the urgent need for advanced microbiological tools [11]. In recent years, the advancement of Next-Generation Sequencing (NGS) has offered rapid culture-independent diagnostics to overcome multiple steps of screening and identification of microorganisms [12]. NGS also provides a comprehensive characterization of microbial communities and diversity, offering a transformative tool that can unlock new opportunities for enhancing food-pathogen control [13]. These insights are critical for managing microbiological risks and detecting emerging foodborne pathogens in seafood-processing environments [14,15].

The wet surfaces of seafood-processing facilities host diverse microbiota, including pathogenic and potentially beneficial organisms. Research suggests that beneficial bacteria in these environments can aid in controlling foodborne pathogens, supporting a bio-economical approach to food safety management [16]. However, NGS applications in seafood processing remain limited, particularly in Malaysia [17]. This study addresses this gap by examining the microbiome diversity in FSMS-certified and -uncertified seafood factories. By integrating NGS, this dual approach of analyzing both culturable and unculturable microorganisms provides valuable insights into FSMS implementation and its effect on pathogen control.

This study’s objective was to compare microbiome diversity in FSMS-certified and uncertified seafood factories to assess microbiological contaminations and enhance food safety practices. This research offers a rapid and culture-independent NGS tool for the identification of food safety-related microorganisms that is crucial for demonstrating the importance of adopting NGS in FSMS practices in mitigating foodborne pathogen risks and contributing to improved food safety and industry sustainability instead of the traditional method of culturable microorganism identification. This study was conducted as part of a pilot scale testing of a newly proposed framework to assist the food safety efforts for audits and FSMS certification surveillance.

## 2. Materials and Methods

### 2.1. Selection of Seafood Factories

Six seafood factories in Penang, Malaysia, processing a range of products, including fillets, surimi and shrimp dim sum, were selected based on voluntary participation. The processing environments of these factories were studied and categorized into key production steps: receiving raw materials, storage, processing and finished-product storage. The selected factories were evaluated based on their compliance with Food Safety Management System (FSMS) practices, which encompass a comprehensive approach involving infrastructure design, process management and documentation, all of which are verified and audited by food safety authorities.

Three factories were designated as the “certified” group, indicating adherence to the national Food Hygiene Regulations 2009, concerning materials, facilities and equipment. These factories are monitored by district food safety authorities, who conduct quarterly microbiological assessments of ice, water, drug residues and seafood organoleptic properties [18]. They are also subject to annual audits by competent authorities, with technical reports provided to guide corrective actions for any non-conformances identified during FSMS certification. The “certified” factories (C, E and F) hold one or more certifications, including Makanan Selamat Tanggungjawab Industri (MeSTI), Hazard Analysis Critical Control Point (HACCP), Food Safety System Certification (FSSC) 22000, British Retail Consortium Global Standards (BRCGS), Best Aquaculture Practice (BAP), Good Manufacturing Practice (GMP) and Veterinary Health Mark (VHM). The remaining three factories, designated as the “uncertified” group (A, B and D), have no FSMS certifications and do not meet the requirements for certification. All “certified” factories have successfully entered export markets, supplying countries across Asia, Europe, North America, Australia and/or South America. In contrast, the “uncertified” factories market their seafood products exclusively within the domestic market. A comparison of the characteristics of the “certified” and “uncertified” seafood factories is presented in Table 1.

A total of 210 environmental swab samples were collected using Environmental Scrub Samplers (ESS, 3M™, St. Paul, MN, USA) from critical sampling locations (CSLs) in all consenting seafood factories during April and May 2022. Table 2 shows the specific seafood products produced in each factory. Among the “uncertified” factories, Factory A produces shrimp dim sum; Factory B produces fish balls; and Factory D specializes in fillets made from salted, broiled mackerel. In comparison, within the “certified” group, Factory C also produces shrimp dim sum, Factory F produces fish balls and Factory E focuses on frozen fillets made from red snapper.

### 2.2. Traditional Methods of Diagnostic

Table 2 details the five sampling sites, which included two direct food-contact sites, one adjacent food-contact site, and one each from the floor and drain areas.

- Site 1 (direct food contact): Utensils such as trays, mixer blades, bowls used for grinding, brining tanks and conveyor belts for descaling.
- Site 2 (direct food contact): Utensils such as racks, trays, bowls for mixing, net scoops for fish handling and tables for degutting.
- Site 3 (adjacent food contact): Tables and machines used for forming, salting and rinsing.
- Site 4: Factory floors.
- Site 5: Drains.

Swabbing was performed during active seafood processing using a zigzag scrubbing motion, with 10 horizontal and 10 vertical strokes, followed by a 90-degree rotation to change direction [19]. Two ESS swabs were collected per sampling site for traditional culturing methods and analysis. Swabs were placed in sterile Whirl-Pak^®^ bags (Pleasant Prairie, Wisconsin, USA) and maintained under cold chain conditions in the range between 0 and 4 °C for traditional analysis. All samples were transported to the laboratory and analyzed within 24 h.

Traditional methods were employed to identify culturable microorganisms in accordance with the International Organisation for Standardisation (ISO) standards for detecting and enumerating foodborne pathogens. These included *Bacillus cereus*, *Listeria monocytogenes*, *Salmonella* spp., *Shigella* spp., *Vibrio cholerae*, *Vibrio parahaemolyticus* and *Vibrio vulnificus* [20,21,22,23,24]. Samples were homogenized, serially diluted, inoculated, and incubated using specific enrichment broths and selective agars tailored to each pathogen as follows:

1. *Bacillus cereus*: Mannitol Egg Yolk Polymyxin agar (Oxoid, Hampshire, UK) and Mannitol Phenol Deoxycholate agar (Oxoid, Hampshire, UK).
2. *Listeria monocytogenes*: Fraser Broth and Oxford Agar (Oxoid, Hampshire, England.).
3. *Salmonella* spp.: Buffered Peptone Water (Oxoid, Hampshire, UK) and Rappaport-Vassiliadis Soy Peptone (Merck, Darmstadt, Germany).
4. *Shigella* spp.: *Shigella* broth with novobiocin (HiMedia, Mumbai, India), Xylose Lysine Deoxycholate agar (Oxoid, Hampshire, UK), MacConkey agar (Oxoid, Hampshire, UK), Hektoen Enteric agar (Oxoid, Hampshire, UK) and nutrient agar (Merck, Darmstadt, Germany).
5. *Vibrio* spp. (V. *cholerae*, V. *parahaemolyticus*, V. *vulnificus*): Alkaline Peptone Water (Oxoid, Hampshire, UK), Selenite F Broth (HiMedia, Mumbai, India) and Thiosulfate–Citrate–Bile Salts–Sucrose agar (Merck, Darmstadt, Germany).

Following incubation, biochemical confirmation tests were conducted to verify the identity of the isolates. Additionally, serological confirmation of foodborne pathogens was performed to ensure accuracy in detection [25,26]. The results from the traditional method are presented in Section 3.1, with discussions in the same section.

### 2.3. Next-Generation Sequencing Method

#### 2.3.1. Sampling Procedures

Sampling procedures for the Next-Generation Sequencing (NGS) method were similar to those described for the traditional method in Section 2.1. The five sampling sites, swabbing motions and type of swab used, and Environmental Scrub Samplers (ESS, 3M™) remained the same, as did the storage in sterile Whirl-Pak^®^ bags (Pleasant Prairie, WI, USA). The primary differences were the number of swabs collected and the cold chain requirements. For NGS analysis, five ESS swabs were collected from each sampling site within each factory belonging to the different certification groups. The swabs were placed in sterile Whirl-Pak^®^ bags (Pleasant Prairie, WI, USA) and maintained under a cold chain condition of below 0 °C. All samples were transported to the laboratory and analyzed according to the NGS workflow outlined in Section 2.3.2 and Section 2.3.3.

#### 2.3.2. NGS Workflow

The Next-Generation Sequencing (NGS) workflow integrated both wet and dry lab procedures to comprehensively analyze microbial diversity. The wet lab steps involved DNA extraction, quality control, polymerase chain reaction (PCR) amplification and amplicon sequencing. For DNA extraction, five ESS samples from each site per factory were pooled, and genomic DNA (gDNA) was extracted using the FastDNA^TM^ Spin Soil Kit (MP Biomedicals LLC, Solon, OH, USA). DNA purity and concentration were assessed using a spectrophotometer (Implen NanoPhotometer^®^ N60/N50) (Implen GmbH, Munich, Germany) and quantified with an Invitrogen Qubit^®^ 2.0 fluorometer (ABP Biosciences, Beltsville, MD, USA). The size of the gDNA was further evaluated through agarose gel electrophoresis to ensure its suitability for downstream applications. PCR amplification focused on the 16S rRNA genes, which are highly variable sequences known as hypervariable regions. These regions exhibit significant variability between different bacterial species, making them ideal for taxonomic identification [27]. To amplify these regions, locus-specific bacterial primers were designed as follows:

- Amplicon Primer, Bacterial 16S V3-V4 (5′ to 3′).
- Forward primer (16S V3-V4): CCTACGGGNGGCWGCAG.
- Reverse primer (16S V3-V4): GACTACHVGGGTATCTAATCC.

The amplicon PCR reaction was conducted using REDiant^®^ 2x PCR Master Mix (1st Base, Singapore) to amplify the bacterial 16S rRNA gene, specifically targeting the V3 and V4 regions. Locus-specific sequence primers with overhang sequences were used, and amplification was conducted with KOD-Multi & Epi^TM^ polymerase (Toyobo, Japan). The PCR thermal cycler program included an initial denaturation at 95 °C for 3 min, followed by 25 cycles of denaturation at 85 °C for 30 s, annealing at 55 °C for 30 s and extension at 72 °C for 30 s, with a final extension at 72 °C for 5 min and a hold at 5 °C. For quality control, the size of DNA amplicons was assessed using the Agilent Bioanalyzer 2100 System with the Agilent DNA 1000 kit (Agilent Technologies, Inc., Santa Clara, CA, USA). Amplicon sequencing was then performed using the Illumina^®^ MiSeq^®^ sequencer (Illumina, Inc., San Diego, CA, USA) with the 2 × 300 bp MiSeq^®^ V3 reagent kit [28,29].

#### 2.3.3. Bioinformatics and Data Analysis

The bioinformatics and data analysis, comprising the dry lab work, focused on processing the V3–V4 hypervariable regions of 16S rRNA sequencing data generated by the MiSeq^®^ platform, employing QIIME2 for comprehensive analysis [30,31]. Primer sequences were trimmed with CUTADAPT (version 4.1), and untrimmed sequences were excluded from the dataset [32]. Sequence merging was performed using VSEARCH (version 2.21.1), followed by denoising to generate amplicon sequence variants (ASVs) via the DADA2 plugin (version 1.22.0) [33,34]. Phylogenetic analysis involved sequence alignment using Multiple Alignment using Fast Fourier Transform (MAFFT) and tree construction with FastTree (version 2.2.10) [35,36]. Taxonomic classification was carried out using a Naive-Bayes classifier trained on the SILVA 1.32 database, implemented through the q2-classifier plugin in QIIME2 (version 2022.8.0) [37,38]. Chloroplast and mitochondrial ASVs were removed, and the data were rarefied to 12,426 reads per sample for downstream analysis. The final feature table, with taxonomic assignments confirmed at a 70% confidence threshold, was exported for further examination.

Alpha and beta diversity metrics, taxonomic comparisons and Analysis of Composition of Microbiomes (ANCOM) were conducted within QIIME2 [30,31]. Alpha diversity, which measures within-sample diversity, was assessed using indices such as observed ASVs, Faith’s Phylogenetic Diversity (PD), and evenness indices like the Simpson and Shannon indices. Statistical significance of these metrics was evaluated using the Kruskal–Wallis test [39,40]. Beta diversity, which quantifies variation between samples, was analyzed using metrics such as Bray–Curtis, Jaccard, unweighted UniFrac and weighted UniFrac. Results were visualized through Principal Coordinate Analysis (PCoA) plots, and differences between certification groups were statistically tested using the PERMANOVA test via the Adonis function at a 5% significance threshold [41,42].

ANCOM was employed to identify differentially abundant taxa between certification groups, leveraging the W-statistic to reduce false discoveries and reveal significant taxonomic differences [43]. Data visualization included heatmaps displaying the 30 most abundant genera and pie charts illustrating the prevalence of lactic acid bacteria (LAB) by certification status. Visualizations were generated using Python libraries: Matplotlib and Seaborn [31,44].

## 3. Results and Discussions

### 3.1. Culturable Pathogens Using Traditional Method

Pathogen analysis presented in Table 3 revealed significant differences between “certified” and “uncertified” seafood-processing facilities. In “uncertified” factories (except Factory D), *Escherichia coli*, *Salmonella* spp., *Listeria monocytogenes* (LM) and *Bacillus cereus* were detected at various key sites, including trays, floors and drains, with distinct serotypes and biofilm-forming abilities. Factory A exhibited notably high contamination levels, with *Salmonella Hindmarsh* found on prawn pressing racks, potentially due to the use of non-food-grade materials like camel-hair ropes [45]. *Listeria monocytogenes* was isolated exclusively from “uncertified” Factory A, persisting on food-contact surfaces despite routine cleaning and sanitation. This persistence suggests structural deficiencies, such as cracked and porous tiles, which facilitate biofilm formation [46]. Additionally, *Salmonella Weltevreden* detected in drain samples highlighted sanitation lapses linked to inadequate infrastructure. Factors such as poor equipment maintenance, lack of control over personnel movement and insufficient cleaning frequency likely to have contributed to pathogen survival and biofilm growth in these facilities [47]. The persistence of *Listeria* and *Salmonella* biofilms on damaged or unsanitary surfaces reinforces the critical need for robust sanitation protocols and FSMS certification to reduce contamination risks [48].

Poor environmental conditions in “uncertified” factories, including cracked floors, porous surfaces and stagnant water, further supported biofilm formation. Irregular and damaged surfaces create microhabitats that shield bacteria from cleaning agents, allowing biofilms to develop and persist over time [49] Additionally, stagnant water provides an optimal medium for bacterial adhesion and biofilm maturation, particularly for waterborne pathogens like *Salmonella* and *Listeria monocytogenes* [46,50,51]. Biofilms act as protective barriers for pathogens, rendering them resistant to sanitizers and facilitating persistent contamination [46]. In “uncertified” Factory B, *Salmonella Bareilly* and *Salmonella Braenderup* were detected on the floor and drain both before and after cleaning, indicating biofilm establishment due to inadequate maintenance. The co-presence of *Escherichia coli* and *Bacillus cereus* highlighted significant hygiene lapses, creating optimal conditions for biofilm-forming pathogens. Research demonstrates that *Salmonella* biofilms can thrive on common surfaces, such as stainless steel and high-density polyethylene (HDPE), which are challenging to sanitize in the absence of FSMS protocols [52]. Furthermore, Factory B’s structural deficiencies, such as open doors permitting pest entry, illustrate how poor facility design, and high renovation costs hinder FSMS implementation and exacerbate contamination risks. These findings underscore that the absence of certified FSMS in “uncertified” facilities enables pathogens to establish persistent reservoirs, raising serious food safety concerns [53].

The lack of structural integrity and inadequate hygiene practices in food-processing facilities characterized the “uncertified” factories. Poor FSMS implementation in “uncertified” Factories A and B was evident in their unhygienic conditions, which facilitated foodborne pathogen contamination and recontamination within the processing environment. The detection of pathogens such as *Escherichia coli*, *Salmonella* spp., *Listeria monocytogenes* and *Bacillus cereus* highlights critical sanitation gaps and structural deficiencies, including cracked tiles and the use of low-quality materials like camel-hair ropes [45,46]. These factors likely contributed to persistent biofilms, enabling the survival of pathogens such as *Listeria monocytogenes* and *Salmonella Weltevreden*. This aligns with previous studies, which indicate that inadequate cleaning and maintenance practices are major contributors to biofilm formation in food-processing environments [47]. The absence of stringent controls, a hallmark of well-implemented FSMS, further exacerbated contamination risks. Sanitary design and construction, including smooth and impervious surfaces and proper site preparation, are critical for preventing biofilm formation and contamination. However, these principles were often neglected in “uncertified” factories, resulting in compromised hygiene practices and increased food safety hazards [54,55].

The pathogen analysis revealed significant differences in microbiological safety between “certified” and “uncertified” seafood-processing facilities, emphasizing the impact of FSMS implementation on contamination control. Inadequate seafood handling practices, ranging from improper processing procedures to cross-contamination via pests or handlers, significantly contributed to contamination risks in “uncertified” factories [56,57]. In contrast, “certified” factories implemented hygienic design principles, ensuring proper site selection, zoning and controlled workflows to minimize food safety hazards. A comparison between “certified” Factories C and F, which produced similar shrimp dim sum and fish ball products as “uncertified” Factories A and B, demonstrated the effectiveness of FSMS. The barrier to the FSMS adoption can be traced to the smaller size of the latter and their lack of resources, training and food safety culture, all critical to ensuring commitment in FSMS implementation [58,59]. The absence of foodborne pathogens in “certified” Factories C and F underscored their robust FSMS implementation, which successfully mitigated contamination risks and ensured product safety [3]. This stark contrast emphasized the critical role of FSMS in maintaining food safety in seafood-processing environments.

Although traditional methods effectively identified specific foodborne pathogens and highlighted the critical role of FSMS in reducing microbiological contamination, their reliance on culturable microorganisms and target-specific detection methods limits their ability to capture the full microbial diversity within seafood-processing environments. As shown in Table 3, traditional methods identified only culturable microorganisms, failing to reveal the broader microbiome diversity, including the unculturable species present on swabbed surfaces. This limitation creates critical gaps in understanding microbial interactions, particularly the role of beneficial bacteria in food safety.

### 3.2. Microbiome in Seafood Factories Measured Using NGS Method

**Figure 1:**
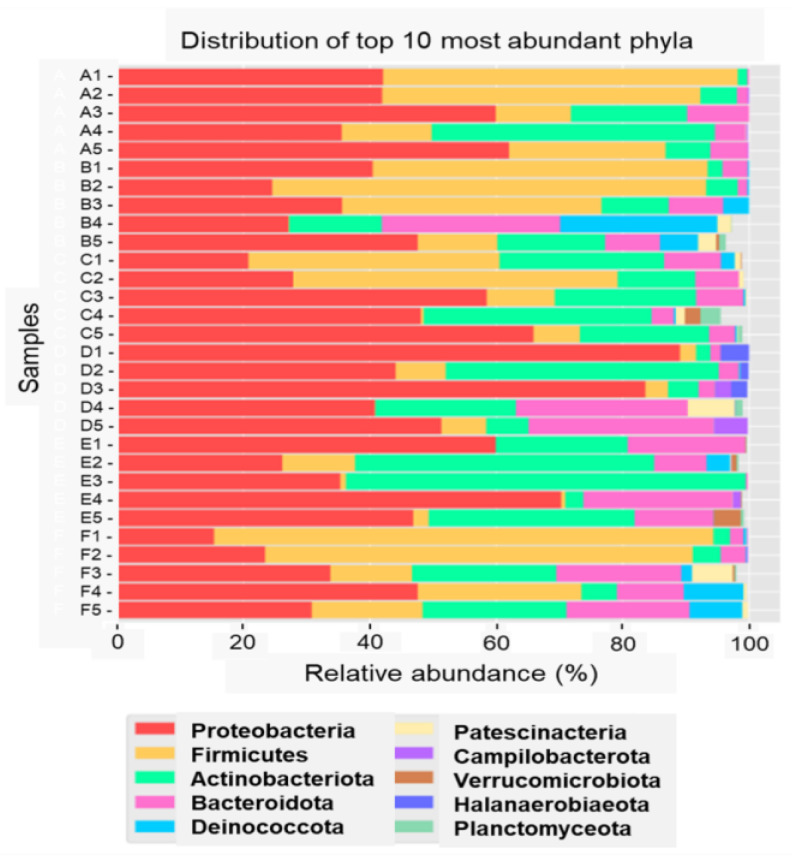
Distribution of the top 10 most abundant phyla (Proteobacteria, Firmicutes, Actinobacteriota, Bacteroidota, Deinococcota, Patescibacteria, Campilobacterota, Verrucomicrobiota, Halanaerobiaeota and Planctomycetota). Sampling sites are labelled by factory identity (A–F) and site number, with Sites 1 and 2 representing Zone 1 (direct food contact); Site 3 representing Zone 2 (adjacent area); and Sites 4 and 5 representing Zone 3 (floor and drain, respectively).

Using Next-Generation Sequencing technology with 16S rRNA amplicon sequencing, this study identified 4100 amplicon sequence variants (ASVs) across 12,947,858 reads, with individual sample reads ranging from 160,057 to 297,076. Rarefaction curve analysis confirmed that the sequencing depth was sufficient to capture the full diversity of the microbiome community, ensuring comprehensive coverage. Analysis of 30 environmental samples from six seafood-processing factories revealed the top 10 most abundant phyla: Proteobacteria, Firmicutes, Actinobacteriota, Bacteroidota, Deinococcota, Patescibacteria, Campilobacterota, Verrucomicrobiota, Halanaerobiaeota and Planctomycetota, as illustrated in Figure 1.

Among these, Proteobacteria (44.56%), Firmicutes (22.76%), Actinobacteriota (18.28%) and Bacteroidota (9.59%) emerged as the dominant phyla, highlighting their significant presence across seafood-processing environments. The microbial composition varied significantly between FSMS “certified” and “uncertified” groups, underscoring the influence of FSMS implementation on microbiome diversity. “Certified” facilities exhibited a relatively balanced microbial composition, with Proteobacteria (20.36%), Firmicutes (10.94%), Actinobacteriota (11.41%) and Bacteroidota (5.01%). In contrast, the “uncertified” facilities demonstrated higher proportions of Proteobacteria (24.20%) and Firmicutes (11.81%) but lower levels of Actinobacteriota (6.87%) and Bacteroidota (4.58%). The consistent presence of Proteobacteria, Actinobacteriota and Bacteroidota across all samples underscores their adaptability and biofilm-forming potential, while Firmicutes, detected in 96.66% of the samples, signifies their substantial prevalence despite slight variations across facilities. Proteobacteria, the most dominant phylum, were notable distributed across all processing sites: 20.04% and 14.08% on direct-contact surfaces (Sites 1 and 2), 22.95% in adjacent areas (Site 3), 20.15% on the floor (Site 4) and 22.78% in the drain (Site 5). This consistent presence highlights Proteobacteria’s role as spoilage microorganisms, contributing to biofilm formation and posing significant food safety risks. The higher prevalence of Proteobacteria in “uncertified” facilities reflects weaknesses in cleaning and sanitization practices, which exacerbate contamination risks. This aligns with findings from previous studies, which report that Proteobacteria, Bacteroidetes and Actinobacteria decrease with improved hygiene conditions [60].

These Next-Generation Sequencing findings demonstrate the richness and complexity of microbiome communities in seafood factories which traditional pathogen-specific methods cannot fully capture. The high relative abundance of Proteobacteria, known for their adaptability and biofilm-forming capacity, highlights their pivotal role in seafood-processing environments. Supporting these findings, previous studies identified Proteobacteria and Firmicutes as the primary phyla in fish-processing environments [61]. Additionally, environmental studies indicate that Proteobacteria and Bacteroidota are more prevalent in seafood environments compared to the meat industry, where Actinobacteriota and Firmicutes dominate [62,63]. These observations suggest that microbial spoilage in seafood processing may arise from raw material contamination or cross-contamination with meat ingredients, particularly in facilities producing mixed products like shrimp dim sum.

The substantial presence of Firmicutes, including lactic acid bacteria (LAB) genera, suggests a potential protective function against pathogens, particularly in “certified” facilities. These findings emphasize the importance of robust FSMS protocols in shaping microbial communities to enhance food safety. The differential distribution of Proteobacteria, Actinobacteriota, Bacteroidota and Firmicutes between “certified” and “uncertified” facilities highlights how enhanced sanitation measures in “certified” facilities contribute to a lower prevalence of spoilage microorganisms and improved food safety outcomes. Overall, this study demonstrates the critical role of microbiome diversity analysis in assessing FSMS effectiveness and optimizing seafood-processing environments.

**Figure 2:**
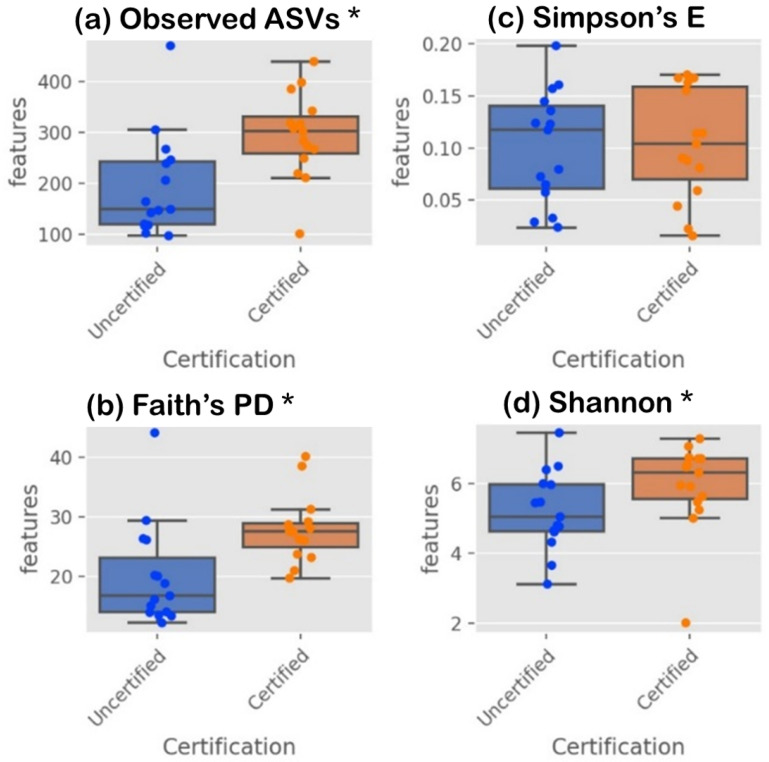
Alpha diversity indices for comparing species richness and evenness between two certification groups using Kruskal–Wallis’s test. (**a**) Observed ASVs and (**b**) Faith’s Phylogenetic Diversity represent the ASV abundance and richness; and (**c**) Simpson and (**d**) Shannon indices reflect ASV diversity and evenness. Asterisks (*) indicate significant differences based on the Kruskal-Wallis’s test (*p* < 0.05).

The alpha diversity boxplots in Figure 2 illustrate the phylogenetic diversity (PD) indices, which indicate greater species richness, alongside Shannon and Simpson indices, which reflect microbiome diversity within samples. Alpha diversity analysis revealed significant differences in microbial richness and diversity across samples, with notable p-values (*p* < 0.05) observed for ASVs (*p* = 0.0036, H = 8.43) and Faith’s PD (*p* = 0.0026, H = 9.04), indicating greater species richness in certain groups. The Shannon index also showed significance (*p* = 0.032, H = 4.56), highlighting differences in microbial diversity among samples. However, the Simpson index (*p* = 0.221, H = 1.50) showed no significant differences in evenness, suggesting that dominant species were evenly distributed across sample groups. Together, these results underscore the utility of alpha diversity indices in evaluating microbiome variation and richness in the studied environments.

**Figure 3:**
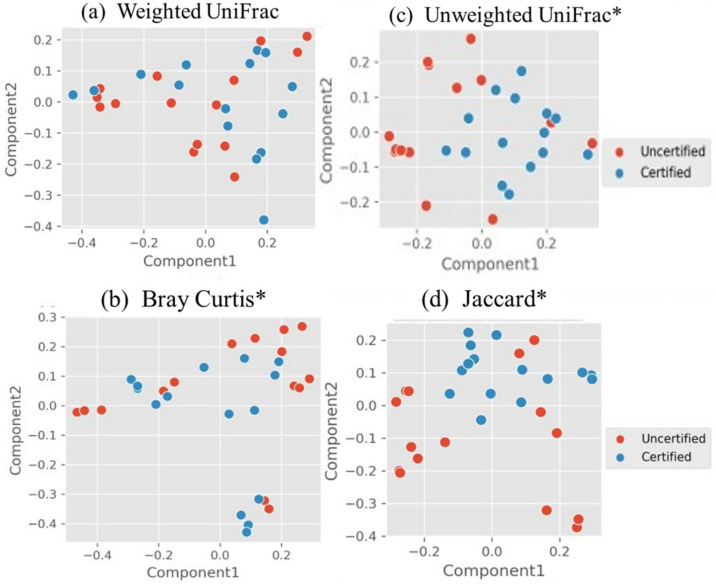
Principal Coordinate Analysis (PCoA) plots of beta diversity indices: (**a**) weighted UniFrac, (**b**) Bray–Curtis, (**c**) unweighted UniFrac and (**d**) Jaccard. Red dots represent the “uncertified” group, and blue dots represent the “certified” group. Asterisks (*) indicate significant differences based on the PERMANOVA test (Adonis, *p* < 0.05).

Beta diversity measures dissimilarity between microbiome communities, providing a complementary perspective to alpha diversity by assessing variations between groups. Figure 3’s Principal Coordinate Analysis (PCoA) plots visualize these differences, with quantitative indices such as weighted UniFrac and Bray–Curtis incorporating species abundance and shared taxa, and unweighted UniFrac and Jaccard focusing solely on species presence or absence. PERMANOVA analysis revealed significant differences in beta diversity for Bray–Curtis (*p* = 0.001, F = 0.002), unweighted UniFrac (*p* = 0.001, F = 0.002) and Jaccard (*p* = 0.001, F = 0.000), indicating substantial variation in relative abundance, phylogenetic dissimilarity and species presence or absence between certification groups. In contrast, no significant difference was observed for weighted UniFrac (*p* = 0.11, F = 0.147), suggesting that abundance-weighted phylogenetic differences are less pronounced. Together, these findings highlight distinct patterns in microbiome community composition between “certified” and “uncertified” groups, driven by differences in species presence, abundance and phylogenetic relationships.

The diversity of microbial communities in food factory environments plays a vital role in mitigating foodborne pathogens. In this study, microbial diversity refers to the variety of different microbial species present in seafood-processing environments. High microbial diversity, as indicated by alpha and beta diversity indices, enhances food safety by fostering beneficial interactions among microbial species. For example, diverse microbiomes can improve biofilm formation and outcompete harmful pathogens, thereby increasing ecosystem stability and resilience [64,65,66]. This stability often acts as a barrier against the immigration of resistant pathogens and provides greater resistance to antimicrobial agents [67,68]. The absence of foodborne pathogens in FSMS-certified facilities, as demonstrated by traditional methods in Section 3.1, aligns with the higher microbial diversity observed in these environments. This suggests that increased microbial diversity contributes directly to improved pathogen suppression and food safety outcomes. Furthermore, variations in microbial diversity along food-processing chains, such as those observed in beef processing, correlate with the presence of safety-relevant genes, emphasizing the importance of monitoring and enhancing microbial diversity to mitigate contamination risks [69].

The implementation of the principles of the Food Safety Management System (FSMS), including cleaning protocols, sanitation measures and adherence to Hazard Analysis Critical Control Point (HACCP) principles, plays a critical role in shaping microbial diversity in food production environments. These systems mitigate microbial hazards by fostering a controlled environment that supports beneficial microbial interactions and enhances microbial biodiversity. For instance, HACCP plans, which involve identifying and monitoring critical control points, are particularly effective in reducing microbiological hazards and ensuring food safety and quality [70]. Facilities with robust FSMS exhibit higher microbial diversity compared to those with inadequate systems, as FSMS enhances monitoring, reduces pathogen prevalence and ensures consistency in identifying key microbial indicators [71]. This higher diversity fosters a balanced ecosystem that suppresses pathogens and minimizes antimicrobial resistance, as reflected in the absence of foodborne pathogens in certified facilities [72]. By creating environments where microbial interactions thrive, FSMS not only ensures food safety but also supports a stable and sustainable production system. These findings highlight the critical importance of FSMS in maintaining microbial diversity, controlling pathogens and reinforcing ecosystem stability in food production.

**Figure 4:**
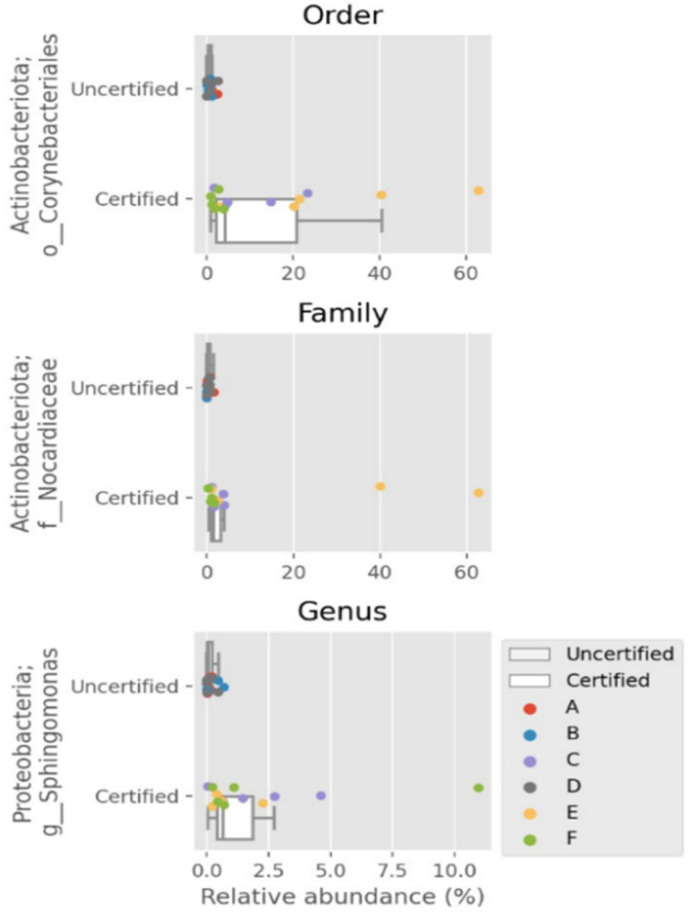
Bar plots of significant relative abundance with ANCOM analysis at order, family and genus levels. Differentially abundant taxa at order level Corynebacteriales from phylum Actinobacteriota; family level Nocardiaceae from phyla Actinobacteriota; and genus level Sphingomonas from phyla Proteobacteria.

The ANCOM analysis identified key differentially abundant taxa between FSMS-certified and -uncertified seafood-processing facilities across multiple taxonomic levels, including order (Corynebacteriales, W = 127), family (Nocardiaceae, W = 198) and genus (Sphingomonas, W = 214) (Figure 4). Corynebacteriales and Nocardiaceae were predominantly associated with certified environments, likely reflecting contributions from human-skin microbiomes, particularly in manual processing areas such as degutting tables [73,74]. Among these taxa, the genus *Sphingomonas* exhibited a significantly higher prevalence in “certified” sites (91.49%) compared to “uncertified” sites (8.51%). Within the “certified” group, Factories C (32.12%), F (45.46%) and E (13.91%) demonstrated substantial representation of this genus, which was distributed across adjacent areas (42.77%), floors (19.81%), drains (15.03%) and direct contact surfaces (13.70%). Known for its biofilm-forming capabilities and pathogen inhibition, *Sphingomonas* plays a critical role in food safety [75,76]. Detected species such as *Sphingomonas panni*, previously isolated from clinical settings, and *Sphingomonas formosensis*, recognized for degrading polycyclic aromatic hydrocarbons, underscore its functional versatility [77,78]. These findings suggest that *Sphingomonas* contributes significantly to the distinct microbiome profiles observed in “certified” facilities. Its role in the biofilm control and pathogen competition highlights how FSMS implementation fosters beneficial communities, supports microbiome diversity and reinforces food safety in seafood-processing environments.

### 3.3. Distinctive Genera in “Certified” and “Uncertified” Seafood Processing Using NGS Method

**Figure 5:**
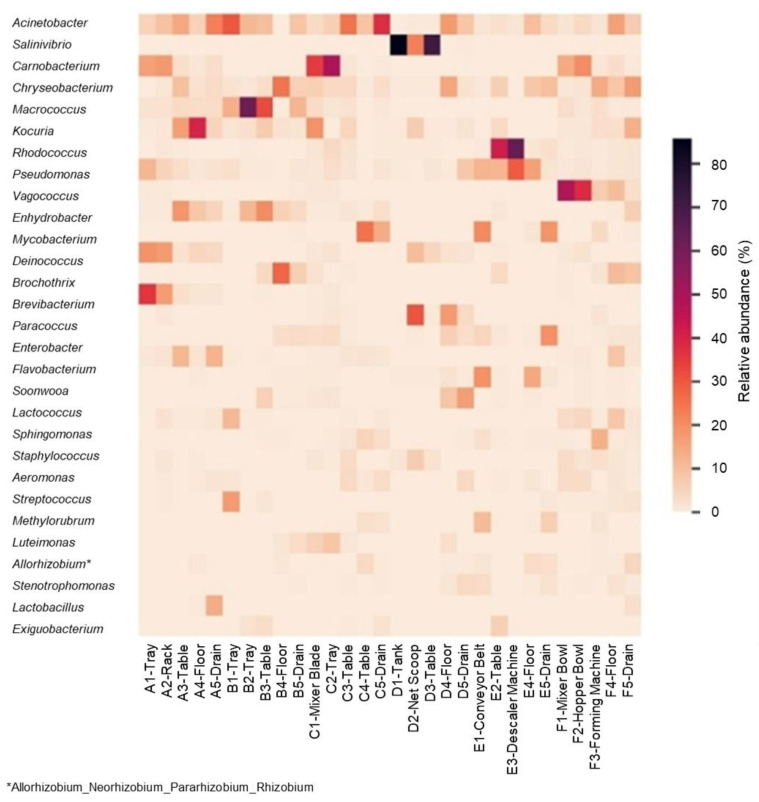
Heatmap of genus-level relative abundance across environmental sites sampled from seafood factories. The color gradient represents relative abundance, with darker shades indicating higher percentages. Sites are labelled by factory identity (A–E) and site number. Site 1 and Site 2 correspond to direct food-contact surfaces; Site 3 to adjacent areas; and Sites 4 and 5 to the floor and drain, respectively.

Figure 5′s heatmap displays the relative abundance of key bacterial genera across 30 sampling sites, highlighting distinct microbial profiles between “certified” and “uncertified” seafood-processing facilities. Seven dominant genera were identified: *Brochothrix*, *Kocuria*, *Macrococcus*, *Carnobacterium*, *Salinivibrio*, *Rhodococcus* and *Vagococcus*. “Uncertified” factories predominantly harbored spoilage-associated genera, including *Brochothrix* and *Kocuria* in Factory A (shrimp dim sum), *Macrococcus* in Factory B (fish ball) and *Salinivibrio* in Factory D (salted broiled mackerel fish). In contrast, “certified” factories were characterized by beneficial genera, such as *Carnobacterium* in Factory C (shrimp dim sum), *Rhodococcus* in Factory E (frozen red snapper) and *Vagococcus* in Factory F (fish ball).

In “certified” facilities, spoilage organisms dominated. *Brochothrix*, a Gram-positive bacterium linked to in fish and meat spoilage, constituted 93.93% of the microbial load on trays (A1) and racks (A2) in Factory A. Known for its association with *Listeria monocytogenes* and the production of spoilage compounds, such as acetoin and diacetyl, its presence signals inadequate hygiene practices [79,80]. *Kocuria*, a biofilm-forming bacterium resistant to desiccation, accounted for 45.91% of microbial loads on tables (A3) and floors (A4), raising concerns about antimicrobial resistance (AMR) and potential transfer of pathogenic gene transfer [62,81]. Similarly, *Macrococcus*, which dominated Factory B with a relative abundance of 80.71%, has been associated with [82,83] methicillin resistance genes and virulence factors [84,85,86]. The presence of these spoilage-associated and AMR-related bacteria heightens the urgent need for stricter sanitation protocols in “uncertified” seafood factories.

Conversely, FSMS-certified facilities exhibited higher levels of beneficial bacteria and an absence of foodborne pathogens (Section 2.1), demonstrating the effectiveness of these systems in fostering safer microbial communities. In Factory C, *Carnobacterium*, a bacterium known for its pathogen inhibition and bio-preservation properties, accounted for 50.79% of the microbial load on mixer blades (C1) and trays (C2) in the shrimp dim sum processing line. Among the identified species, *Carnobacterium maltaromaticum* was detected with a 72% confidence level. Originally isolated from rainbow trout farms, this species inhibits *Listeria monocytogenes* (LM) biofilms for up to five days on stainless-steel surfaces in salmon processing plants [87,88,89]. Additionally, *Carnobacterium* has shown antagonistic effects against *Escherichia coli* (EC) O157:H7 [90]. Factory E had a high prevalence of *Rhodococcus* (81.77%), a genus known for pathogen competition and biofilm mitigation [91,92]. In Factory F, *Vagococcus*, (70.5%) was abundant on mixing bowls (F1) and hopper bowls (F2), contributing to food safety through bacteriocin production [93]. The presence of beneficial microorganisms underscores the role of FSMS certification in promoting competitive exclusion of pathogens and enhancing bio-preservation.

Findings from Next-Generation Sequencing technology showcase how FSMS certification fosters microbial environments that enhance product safety through bio-preservation and pathogen suppression. The stark contrast between spoilage organisms in “uncertified” facilities and beneficial bacteria in “certified” settings underscores the importance of stringent FSMS protocols in mitigating microbial hazards, including antimicrobial resistance (AMR) and biofilm formation [87,94]. “Uncertified” facilities also harbored foodborne pathogens, including genera such as *Escherichia* and *Bacillus*, aligning with studies showing these microorganisms as carriers of key resistance determinants [95]. Research suggests that suboptimal daily cleaning practices can contribute to the spread of antimicrobial-resistance genes in food-processing environments, further emphasizing the risks associated with poor FSMS implementation [95]. These findings reinforce the effectiveness of FSMS-certified systems in shaping microbial diversity and safeguarding seafood-processing environments.

### 3.4. Identification of Lactic Acid Bacteria in Seafood Processing

**Figure 6:**
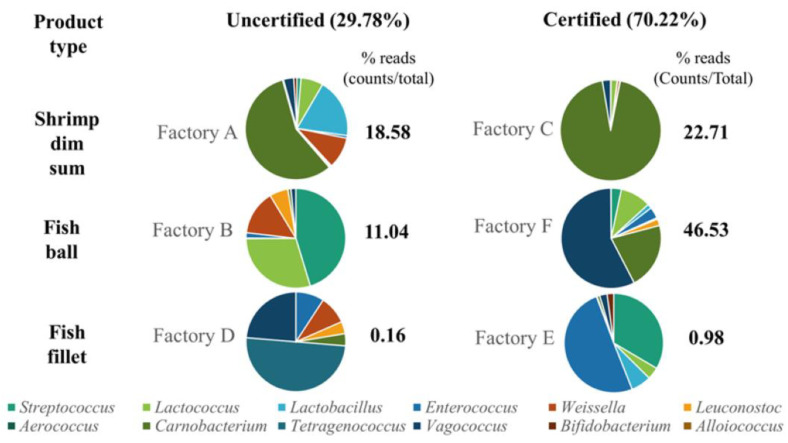
The LAB genera present at “uncertified” and “certified” groups of seafood factories were Streptococcus, Lactococcus, Lactobacillus, Enterococcus, Weissella, Leuconostoc, Aerococcus, Carnobacterium, Tetragenococcus, Vagococcus, Bifidobacterium and Alloiococcus.

The Next-Generation Sequencing (NGS) technology assisted in identification of lactic acid bacteria (LAB), which traditional methods are unable to identify. Figure 6 illustrates LAB distribution in both “certified” and “uncertified” seafood factories. Identified LAB genera included *Aerococcus*, *Alloiococcus*, *Bifidobacterium*, *Carnobacterium*, *Enterococcus*, *Lactobacillus*, *Lactococcus*, *Leuconostoc*, *Streptococcus*, *Tetragenococcus*, *Vagococcus* and *Weissella*. LAB accounted for 70.22% (32,679 reads) of the total microbiota in “certified” factories, compared to 29.78% (13,856 reads) in “uncertified” factories. Specifically, the “certified” factories, Factories C, F and E, exhibited LAB percentages of 22.71%, 46.53% and 0.98%, respectively, while “uncertified” factories, Factories A, B and D, showed LAB percentages of 18.58%, 11.04% and 0.16%, respectively. These findings indicate that FSMS implementation in seafood factories enhances the presence of beneficial LAB strains, which may contribute to improved food safety and bio-preservation through their antimicrobial and antibiofilm mechanisms. The contrasting microbiome profiles observed between “certified” and “uncertified” factories support the hypothesis that robust FSMS protocols reduce contamination risks by promoting beneficial microbial communities. In “certified” factories, the higher abundance of LAB and the absence of foodborne pathogens reflect the effectiveness of sanitation protocols in controlling microbial hazards and fostering beneficial bacteria. These findings are consistent with research demonstrating that FSMS protocols, including HACCP and Good Manufacturing Practices (GMP), mitigate contamination risks and promote bio-preservation [48,96].

Lactic acid bacteria (LAB) are a diverse group of Gram-positive bacteria within the phylum Firmicutes, class Bacilli and order Lactobacillales. Key genera include *Lactobacillus*, *Lactococcus*, *Streptococcus*, *Enterococcus*, *Leuconostoc*, *Carnobacterium*, *Oenococcus*, *Pediococcus*, *Tetragenococcus*, *Vagococcus* and *Weissella* [97]. LAB are commonly found in fermented foods, plants and the human body, and they are renowned for their preservative properties, including protection against spoilage microorganisms and inhibition of foodborne pathogens [98]. Beyond preservation, LAB show potential as biocontrol agents in food processing, particularly in managing pathogens on processing surfaces through the antibiofilm activity of their ribosomal synthesized antimicrobial peptides [99].

Notably, “certified” factories exhibited higher levels of LAB genera, such as *Alloiococcus*, *Carnobacterium*, *Enterococcus*, *Lactococcus*, *Leuconostoc* and *Vagococcus*. These genera are commonly associated with the natural microflora of seafood in fresh or marine environments [100]. In contrast, “uncertified” factories showed greater occurrences of *Aerococcus*, *Bifidobacterium*, *Lactobacillus*, *Streptococcus*, *Tetragenococcus* and *Weissella*. The prevalence of LAB in “certified” facilities underscores their role in enhancing microbiological safety through competitive exclusion, biofilm inhibition and antimicrobial metabolite production, which collectively contribute to the suppression of foodborne pathogens. The competitive exclusion mechanism of LAB is crucial in food safety, as these beneficial bacteria outcompete pathogens for adhesion sites and essential nutrients on seafood-processing surfaces, limiting pathogen colonization [98]. This is particularly important in FSMS-certified factories, where strict hygiene controls foster an environment that supports LAB proliferation while reducing external contamination risks [101]. Moreover, LAB form pre-established biofilms that serve as protective barriers, preventing pathogens attachment and limiting biofilm formation by competing for available surface space [99].

LAB strains from seafood environments are particularly effective in antagonizing foodborne pathogens through the production of antimicrobial metabolites, including organic acids, hydrogen peroxide and bacteriocins [98]. These metabolites disrupt pathogens by targeting cell membranes, inhibiting biosynthesis and competing for essential resources [99]. Organic acids, such as lactic acid and acetic acid, lower the pH of the surrounding environment, creating inhospitable conditions for pathogens like *Listeria monocytogenes* and *Escherichia coli* [102,103,104]. Additionally, hydrogen peroxide disrupts bacterial membranes and induces oxidative stress, leading to cell damage and the inhibition of pathogenic bacteria [105]. The production of bacteriocins (e.g., nisin and pediocin) further enhances LAB’s antimicrobial activity by targeting and disrupting the membrane integrity of Gram-positive pathogens, preventing their growth and replication [106]. In addition to their antimicrobial properties, LAB function as biofilm inhibitors by producing biosurfactants, which weaken bacterial adhesion forces and disrupt pre-existing biofilms [107]. These biofilm-disrupting properties are particularly advantageous in seafood processing, where surface contamination poses a major risk to food safety.

The presence of naturally occurring LAB on seafood-processing surfaces in FSMS-certified factories reduces pathogen biofilms, leading to fewer contamination hotspots compared to “uncertified” facilities, which suffer from poor infrastructure and inadequate sanitation, resulting in higher biofilm-associated bacterial loads [108,109]. FSMS-certified facilities exhibit significantly lower pathogen presence due to robust microbiological hazard controls, including prerequisite programs (PRPs) and strict sanitation protocols outlined in Good Manufacturing Practices (GMP). The consistent implementation of FSMS protocols, such as HACCP certification, ensures compliance with infrastructure design, processing control and sanitation measures, creating an environment that fosters beneficial microbial growth and pathogen suppression. As a result, LAB thrive as natural biocontrol agents, helping to mitigate foodborne risks [110]. The nutrient-rich environment of seafood-processing facilities further supports LAB proliferation, contributing to microbiological stability and improved food safety outcomes, particularly in “certified” factories, where stringent hygiene standards are maintained [111]. In contrast, “uncertified” factories, where traditional microbiological analysis (Section 3.1) confirmed the presence of foodborne pathogens, lack effective sanitation measures, allowing harmful bacteria to persist. Specific LAB strains have demonstrated strong inhibitory effects against *Listeria* spp. and *Escherichia coli*, preventing their biofilm formation and contamination of food surfaces [104,112]. FSMS-certified factories leverage these properties through HACCP-certified sanitation and monitoring protocols, which suppress microbiological hazards while promoting beneficial microbial growth [96].

## 4. Conclusions

The traditional method is limited to detecting only culturable microorganisms, thereby overlooking the diversity of unculturable microbiota. While effective in identifying specific foodborne pathogens, such as the *Escherichia coli*, *Salmonella strains*, *Bacillus cereus* and *Listeria monocytogenes*, in the “uncertified” facilities, these methods fail to capture the broader microbial ecology within Food Safety Management System (FSMS) certification groups. In contrast, Next-Generation Sequencing (NGS) technology, specifically 16S rRNA amplicon sequencing, enables a comprehensive assessment of microbiome diversity, distinguishing “certified” seafood factories from “uncertified” ones. “Certified” seafood factories demonstrated significantly higher alpha and beta diversity metrics, with a greater beneficial microbiota, particularly lactic acid bacteria (LAB) (70.22% vs. 29.78% in “uncertified”). The absence of foodborne pathogens in “certified” facilities underscores the effectiveness of FSMS protocols in fostering competitive exclusion and bio-preservation. Conversely, the higher prevalence of Proteobacteria in “uncertified” factories highlights sanitation deficiencies and increased microbiological risks. Unlike traditional methods, NGS provides a more holistic evaluation of microbial diversity, allowing for the detection of both beneficial and opportunistic bacteria. These findings advocate for the integration of NGS into FSMS certifications surveillance frameworks, enhancing microbial monitoring, food safety management and risk mitigation. By offering superior insights into microbiome composition, NGS supports broader FSMS adoption, improved food safety outcomes and increased marketability of seafood products.

## Figures and Tables

**Table 1:** Characteristics of seafood factories from “certified” and “uncertified” groups selected for investigation of microbiological diversity in processing environment.

Seafood Factories	Year Est.	Years of FSMS Adoption	No. of Workers	FSMS Status	Market	Production Shift	Annual Returns
Uncertified							
A	1995	0	14	None	Domestic	Morning and afternoon	>RM300 k
B	2018	0	15	None	Domestic	Morning	RM300 k
D	2010	1	9	None	Domestic	Morning and afternoon	>RM1.5 m
Certified							
C	2007	7	45	MeSTI	Asia	Morning	>RM1.5 m
E	1985	12	58	HACCP, FSSC22000, BRCGS, BAP	Asia, Europe, North America, Australia	Morning	>RM1.5 m
F	1999	15	20	HACCP, GMP, VHM	Asia, South America	Morning	>RM1.5 m

**Table 2:** Overview of five environmental sampling sites sampled from the six seafood factories for 16S rRNA amplicon sequencing using the NGS method.

Factory	“Uncertified”			“Certified”		
Product	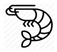 Dim Sum	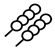 Fish ball	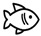 Fish	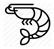 Dim Sum	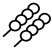 Fish ball	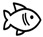 Fish
Sampling Site	A	B	D	C	F	E
1 Direct food contact	Tray	Tray	Tank	Mixer blade	Mixer bowl	Conveyor belt
2 Direct food contact	Rack	Tray	Net scoop	Tray	Hopper bowl	Degutting table
3 Adjacent food contact	Table	Table	Table	Table	Forming machine	Skinning machine
4 Floor	Floor	Floor	Floor	Floor	Floor	Floor
5 Drain	Drain	Drain	Drain	Drain	Drain	Drain

**Table 3:** Culturable isolates’ serovars with foodborne pathogens identified from the traditional method at environmental swab sampling sites of seafood factories from two certification groups with conditions and materials at pre- and post-cleaning.

ID	Sites	Condition and Material	Serovar (Pre-Cleaning)	Serovar (Post-Cleaning)
“Uncertified” seafood factories				
A	1: Prawn-paste tray	Unclean, uneven aluminum surface	*Escherichia coli* ^†^	*Listeria monocytogenes*
	2: Prawn pressing rack	Unclean, HDPE	*Escherichia coli* ^†^, *Salmonella Hindmarsh*	*Escherichia coli* ^†^
	3: Processing table	Unclean, SUS and rope	*Escherichia coli* ^†^, *Salmonella typhimurium*	N/A
	4: Floor	Cracked and porous tiles	N/A	*Listeria monocytogenes*
	5: Drain	Uncovered cement/half-covered SUS	*Salmonella Weltevreden*	*N/A*
B	1: Fish-cake short-forming tray	Unclean, SUS	N/A	N/A
	2: Fish-cake long-forming tray	Unclean, SUS	N/A	*Bacillus cereus*
	3: Forming table	Unclean, SUS	N/A	N/A
	4: Floor	Cracked and porous tiles	*Salmonella Bareilly*	*Bacillus cereus* *Salmonella Braenderup* *Escherichia coli* ^†^
	5: Drain	Uncovered, porous and cracked cement surface	*Salmonella Bareilly*, *Bacillus cereus*	*Bacillus cereus,* *Salmonella Bareilly, Escherichia coli* ^†^,
**D**	1. Brine tank	Polycarbonate	N/A	N/A
	2. Net scoop	SUS and rope	N/A	N/A
	3. Salting table	Unclean, SUS	N/A	N/A
	4: Floor	Epoxy	N/A	N/A
	5. Drain	Half-covered, SUS	N/A	N/A
“Certified” seafood factories				
**C**	1. Prawn-paste mixer blade	SUS	N/A	N/A
	2. Prawn-paste holding tray	PP	N/A	N/A
	3. Hopper bowl	SUS	N/A	N/A
	4. Floor	Tiles	N/A	N/A
	5. Drain	Fully/half-covered, SUS	N/A	N/A
**E**	1. Descaler conveyor belt	PP	N/A	N/A
	2. Degutting and filleting table	SUS	N/A	N/A
	3. Water-jet skinning machine	Rubber and SUS	N/A	N/A
	4. Floor	Epoxy and cement	N/A	N/A
	5. Drain	Half-covered, SUS	N/A	N/A
**F**	1. Fish-paste mixer bowl	Unclean, SUS	N/A	N/A
	2. Hopper bowl	SUS	N/A	N/A
	3. Forming machine	Iron	N/A	N/A
	4. Floor	Epoxy	N/A	N/A
	5. Drain	Half-covered, SUS	N/A	N/A

^†^ *Escherichia coli* serological strains are not of EHEC, Enterohaemorrhagic *Escherichia coli*; EPEC, Enteropathogenic *Escherichia coli*; EIEC, Enteroinvasive *Escherichia coli*; and ETEC, Enterotoxigenic *Escherichia coli* strains.

## Data Availability

The raw data supporting the conclusions of this article will be made available by the authors on request.

## Abbreviations

The following abbreviations are used in this manuscript:

BAP	Best Aquaculture Practice
BRCGS	British Retail Consortium Global Standards
Est.	Established
FSMS	Food Safety Management System
FSSC22000	Food Safety System Certification (FSSC) 22000
GMP	Good Manufacturing Practices
HACCP	Hazard Analysis Critical Control Point
HDPE	high-density polyethylene
k	thousand (10^3^)
m	million (10^6^)
MeSTI	Makanan Selamat, Tanggungjawab Industri
N/A	not applicable
PERMANOVA	Permutational Multivariate Analysis of Variance
PP	polypropylene
pH	potential of hydrogen
RM	Ringgit Malaysia
SUS	stainless steel
VHM	Veterinary Health Mark

## References

[1] FAO FAO Fisheries & Aquaculture—Quality and Safety of Fish and Fish Products. Food and Agriculture Organization of the United Nations. http://www.fao.org/fishery/quality_safety/en.

[2] FAO FAO Fisheries & Aquaculture—Fishery and Aquaculture Country Profiles—Malaysia. FAO. http://www.fao.org/fishery/facp/MYS/en.

[3] Lee J.C., Daraba A., Voidarou C., Rozos G., El Enshasy H.A., Varzakas T. (2021). Implementation of Food Safety Management Systems along with Other Management Tools (HAZOP, FMEA, Ishikawa, Pareto). The Case Study of *Listeria monocytogenes* and Correlation with Microbiological Criteria. Foods.

[4] Fernando Y., Ng H., Walters T. (2015). Regulatory incentives as a moderator of determinants for the adoption of Malaysian food safety system. Br. Food J..

[5] Ahamat H., Manaf N.H.A., Manap N.A., Rahman N.A. (2019). Regulatory Strategies for Facilitating Exports by Microenterprises in Malaysia. Malays. J. Bus. Econ. (MJBE).

[6] Surya T., Jeyasekaran G., Shakila R.J., Sivaraman B., Shalini R., Sundhar S., Arisekar U. (2022). Prevalence of biofilm forming *Salmonella* in different seafood contact surfaces of fishing boats, fish landing centres, fish markets and seafood processing plants. Mar. Pollut. Bull..

[7] Sudagidan M., Ozalp V.C., Öztürk O., Yurt M.N.Z., Yavuz O., Tasbasi B.B., Ucak S., Mavili Z.S., Coban A., Aydin A. (2021). Bacterial surface, biofilm and virulence properties of *Listeria monocytogenes* strains isolated from smoked salmon and fish food contact surfaces. Food Biosci..

[8] Jacxsens L., Kussaga J., Luning P.A., Van der Spiegel M., Devlieghere F., Uyttendaele M. (2009). A Microbial Assessment Scheme to measure microbial performance of Food Safety Management Systems. Int. J. Food Microbiol..

[9] Fathurrahman R.N., Rukayadi Y., Ungku Fatimah U.Z.A., Jinap S., Abdul-Mutalib N.A., Sanny M. (2021). The performance of food safety management system in relation to the microbiological safety of salmon nigiri sushi: A multiple case study in a Japanese chain restaurant. Food Control.

[10] Banerjee G., Agarwal S., Marshall A., Jones D.H., Sulaiman I.M., Sur S., Banerjee P. (2022). Application of advanced genomic tools in food safety rapid diagnostics: Challenges and opportunities. Curr. Opin. Food Sci..

[11] Janekrongtham C., Dejburum P., Sujinpram S., Rattanathumsakul T., Swaddiwudhipong W. (2022). Outbreak of seafood-related food poisoning from undetectable *Vibrio parahaemolyticus*-like pathogen, Chiang Mai Province, Thailand, December 2020. Trop. Med. Int. Health.

[12] Harper S., Counihan K.L., Kanrar S., Paoli G.C., Tilman S., Gehring A.G. (2024). Investigating the Quantification Capabilities of a Nanopore-Based Sequencing Platform for Food Safety Application via External Standards of Lambda DNA and Lambda Spiked Beef. Foods.

[13] Imanian B., Donaghy J., Jackson T., Gummalla S., Ganesan B., Baker R.C., Henderson M., Butler E.K., Hong Y., Ring B. (2022). The power, potential, benefits, and challenges of implementing high-throughput sequencing in food safety systems. Npj Sci. Food.

[14] De Filippis F., Parente E., Ercolini D. (2018). Recent Past, Present, and Future of the Food Microbiome. Annu. Rev. Food Sci. Technol..

[15] Billington C., Kingsbury J.M., Rivas L. (2022). Metagenomics Approaches for Improving Food Safety: A Review. J. Food Prot..

[16] Baráti-Deák B., Mohácsi-Farkas C., Belák Á. (2020). Searching for Antagonistic Activity of Bacterial Isolates Derived from Food Processing Environments on Some Food-Borne Pathogenic Bacteria. Acta Aliment..

[17] Rizal N.S.M., Neoh H.-M., Ramli R., Periyasamy P.R.A.K., Hanafiah A., Samat M.N.A., Tan T.L., Wong K.K., Nathan S., Chieng S. (2020). Advantages and Limitations of 16S rRNA Next-Generation Sequencing for Pathogen Identification in the Diagnostic Microbiology Laboratory: Perspectives from a Middle-Income Country. Diagnostics.

[18] FSQ Fish & Fish Product to the European Union (EU). https://hq.moh.gov.my/fsq/ikan-hasilan-ikan-ke-kesatuan-eropah-eu?.

[19] NSW Food Authority Environmental Swabbing. https://www.foodauthority.nsw.gov.au/sites/default/files/2020-01/environmental_swabbing.pdf.

[20] (2004). Microbiology of Food and Animal Feeding Stuffs—Horizontal Method for the Enumeration of Presumptive *Bacillus cereus*; Colony-Count Technique at 30 °C.

[21] (2017). Microbiology of the Food Chain—Horizontal Method for the Detection and Enumeration of *Listeria monocytogenes* and of *Listeria* spp., Part 1: Detection Method.

[22] (2017). Microbiology of the Food Chain—Horizontal Method for the Detection, Enumeration and Serotyping of Salmonella—Part 1: Detection of Salmonella spp.

[23] (2017). Microbiology of the Food Chain—Horizontal Method for the Determination of *Vibrio* spp.—Part 1: Detection of Potentially Enteropathogenic *Vibrio parahaemolyticus*, *Vibrio cholerae* and *Vibrio vulnificus*.

[24] (2004). Microbiology of Food and Animal Feeding Stuffs—Horizontal Method for the Detection of Shigella spp.

[25] Grimont P.A.D., Weill F.-X. (2007). Antigenic Formulae of the Salmonella Serovars.

[26] Ochman H., Selander R.K. (1984). Standard Reference Strains of *Escherichia coli* from Natural Populations. J. Bacteriol..

[27] Klindworth A., Pruesse E., Schweer T., Peplies J., Quast C., Horn M., Glöckner F.O. (2013). Evaluation of general 16S ribosomal RNA gene PCR primers for classical and next-generation sequencing-based diversity studies. Nucleic Acids Res..

[28] Illumina (2013). 16S Metagenomic Sequencing Library Preparation Preparing 16S Ribosomal RNA Gene Amplicons for the Illumina MiSeq System. http://support.illumina.com/content/dam/illumina-support/documents/documentation/chemistry_documentation/16s/16s-metagenomic-library-prep-guide-15044223-b.pdf.

[29] Illumina (2017). Illumina 16S Metagenomics Sequencing Workflow. Illumina.

[30] Bolyen E., Rideout J.R., Dillon M.R., Bokulich N.A., Abnet C.C., Al-Ghalith G.A., Alexander H., Alm E.J., Arumugam M., Asnicar F. (2019). Reproducible, interactive, scalable and extensible microbiome data science using QIIME 2. Nat. Biotechnol..

[31] Hunter J.D. (2007). Matplotlib: A 2D Graphics Environment. CSE.

[32] Martin M. (2011). Cutadapt removes adapter sequences from high-throughput sequencing reads. EMBnet J..

[33] Rognes T., Flouri T., Nichols B., Quince C., Mahé F. (2016). VSEARCH: A versatile open source tool for metagenomics. PeerJ.

[34] Callahan B.J., McMurdie P.J., Rosen M.J., Han A.W., Johnson A.J.A., Holmes S.P. (2016). DADA2: High-resolution sample inference from Illumina amplicon data. Nat. Methods.

[35] Price M.N., Dehal P.S., Arkin A.P. (2010). FastTree 2—Approximately Maximum-Likelihood Trees for Large Alignments. PLoS ONE.

[36] Katoh K., Standley D.M. (2013). MAFFT Multiple Sequence Alignment Software Version 7: Improvements in Performance and Usability. Mol. Biol. Evol..

[37] Bokulich N.A., Kaehler B.D., Rideout J.R., Dillon M., Bolyen E., Knight R., Huttley G.A., Gregory Caporaso J. (2018). Optimizing taxonomic classification of marker-gene amplicon sequences with QIIME 2’s q2-feature-classifier plugin. Microbiome.

[38] Pedregosa F., Varoquaux G., Gramfort A., Michel V., Thirion B., Grisel O., Blondel M., Prettenhofer P., Weiss R., Dubourg V. (2011). Scikit-learn: Machine Learning in Python. J. Mach. Learn. Res..

[39] Kruskal W.H., Wallis W.A. (1952). Use of Ranks in One-Criterion Variance Analysis. J. Am. Stat. Assoc..

[40] Kers J.G., Saccenti E. (2022). The Power of Microbiome Studies: Some Considerations on Which Alpha and Beta Metrics to Use and How to Report Results. Front. Microbiol..

[41] Anderson M.J. (2001). A new method for non-parametric multivariate analysis of variance. Austral Ecol..

[42] Oksanen J., Guillaume B.F., Friendly M., Kindt R., Legendre P., McGlinn D., Minchin P.R., O’Hara R.B., Simpson G.L., Solymos P.H. Vegan: Community Ecology Package Version: 2.5-6. https://cran.r-project.org/web/packages/vegan/index.html.

[43] Mandal S., van Treuren W., White R.A., Eggesbø M., Knight R., Peddada S.D. (2015). Analysis of composition of microbiomes: A novel method for studying microbial composition. Microb. Ecol. Health Dis..

[44] Waskom M.L. (2021). seaborn: Statistical data visualization. J. Open Source Softw..

[45] Münch S., Braun P., Wernery U., Kinne J., Pees M., Flieger A., Tietze E., Rabsch W. (2012). Prevalence, serovars, phage types, and antibiotic susceptibilities of *Salmonella* strains isolated from animals in the United Arab Emirates from 1996 to 2009. Trop. Anim. Health Prod..

[46] Finn L., Onyeaka H., O’Neill S. (2023). *Listeria monocytogenes* Biofilms in Food-Associated Environments: A Persistent Enigma. Foods.

[47] Fagerlund A., Langsrud S., Møretrø T. (2021). Microbial diversity and ecology of biofilms in food industry environments associated with *Listeria monocytogenes* persistence. Curr. Opin. Food Sci..

[48] Silva A., Silva V., Gomes J.P., Coelho A., Batista R., Saraiva C., Esteves A., Martins Â., Contente D., Diaz-Formoso L. (2024). *Listeria monocytogenes* from Food Products and Food Associated Environments: Antimicrobial Resistance, Genetic Clustering and Biofilm Insights. Antibiotics.

[49] Carrascosa C., Raheem D., Ramos F., Saraiva A., Raposo A. (2021). Microbial Biofilms in the Food Industry—A Comprehensive Review. Int. J. Environ. Res. Public Health.

[50] Vázquez-Sánchez D., Galvão J.A., Oetterer M. (2017). Contamination sources, serogroups, biofilm-forming ability and biocide resistance of *Listeria monocytogenes* persistent in tilapia-processing facilities. J. Food Sci. Technol..

[51] Surya T., Jeyasekaran G., Shakila R.J., Alsalhi M.S., Devanesan S., Sivaraman B., Arisekar U., Pham T.H. (2023). Effect of antibiotics and sanitizers on *Salmonella* biofilms associated with seafood contact surfaces. Microbiol. Res..

[52] Tee X.W., Abdul-Mutalib N.A. (2023). *Salmonella* Biofilm on Food Contact Surfaces and the Efficacy of Chemical Disinfectants: A Systematic Review. Pertanika J. Sci. Technol..

[53] Holah J. (2023). Minimum hygienic design requirements for food processing factories. Hygienic Design of Food Factories.

[54] Marriott N.G., Schilling M.W., Gravani R.B. (2018). Sanitary Design and Construction for Food Processing. Principles of Food Sanitation.

[55] Faille C., Cunault C., Dubois T., Bénézech T. (2018). Hygienic design of food processing lines to mitigate the risk of bacterial food contamination with respect to environmental concerns. Innov. Food Sci. Emerg. Technol..

[56] Brauge T., Mougin J., Ells T., Midelet G. (2024). Sources and contamination routes of seafood with human pathogenic *Vibrio* spp.: A Farm-to-Fork approach. Compr. Rev. Food Sci. Food Saf..

[57] Wan Norhana M.N., Poole S.E., Deeth H.C., Dykes G.A. (2010). Prevalence, persistence and control of *Salmonella* and *Listeria* in shrimp and shrimp products: A review. Food Control.

[58] Dima A., Radu E., Dobrin C. (2024). Exploring Key Barriers of HACCP Certification Adoption in the Meat Industry: A Decision-Making Trial and Evaluation Laboratory Approach. Foods.

[59] Lee J.C., Neonaki M., Alexopoulos A., Varzakas T. (2023). Case Studies of Small-Medium Food Enterprises around the World: Major Constraints and Benefits from the Implementation of Food Safety Management Systems. Foods.

[60] Zhang J., Lu Z., Feng L., Qu D., Zhu J. (2024). Identification of microbial communities and multi-species biofilms contamination in seafood processing environments with different hygiene conditions. Food Microbiol..

[61] Anihouvi D.G.H., Henriet O., Kpoclou Y.E., Scippo M., Hounhouigan D.J., Anihouvi V.B., Mahillon J. (2021). Bacterial diversity of smoked and smoked-dried fish from West Africa: A metagenomic approach. J. Food Process. Preserv..

[62] Nikolaev Y., Yushina Y., Mardanov A., Gruzdev E., Tikhonova E., El-Registan G., Beletskiy A., Semenova A., Zaiko E., Bataeva D. (2022). Microbial Biofilms at Meat-Processing Plant as Possible Places of Bacteria Survival. Microorganisms.

[63] Rodríguez-López P., Rodríguez-Herrera J.J., Cabo M.L. (2020). Tracking bacteriome variation over time in *Listeria monocytogenes*-positive foci in food industry. Int. J. Food Microbiol..

[64] Ross S.R.P.J., Sasaki T. (2024). Limited theoretical and empirical evidence that response diversity determines the resilience of ecosystems to environmental change. Ecol. Res..

[65] Pimm S.L. (1984). The complexity and stability of ecosystems. Nature.

[66] Sadiq F.A., De Reu K., Burmølle M., Maes S., Heyndrickx M. (2023). Synergistic interactions in multispecies biofilm combinations of bacterial isolates recovered from diverse food processing industries. Front. Microbiol..

[67] Palanisamy V., Bosilevac J.M., Barkhouse D.A., Velez S.E., Chitlapilly Dass S. (2023). Shotgun-metagenomics reveals a highly diverse and communal microbial network present in the drains of three beef-processing plants. Front. Cell. Infect. Microbiol..

[68] Klümper U., Gionchetta G., Catão E., Bellanger X., Dielacher I., Elena A.X., Fang P., Galazka S., Goryluk-Salmonowicz A., Kneis D. (2024). Environmental microbiome diversity and stability is a barrier to antimicrobial resistance gene accumulation. Commun. Biol..

[69] Sequino G., Cobo-Diaz J.F., Valentino V., Tassou C., Volpe S., Torrieri E., Nychas G.-J., Ordóñez A.Á., Ercolini D., De Filippis F. (2024). Microbiome mapping in beef processing reveals safety-relevant variations in microbial diversity and genomic features. Food Res. Int..

[70] Eissa R.A.M., Salem M.A., Elsanat S.Y. (2019). Controlling of Potential Hazard in Potato Chips Processing through Food Safety Management System FSMS (ISO 22000). Menoufia J. Food Dairy Sci..

[71] Lacorte G.A., Cruvinel L.A., Ávila M.d.P., Dias M.F., Pereira A.d.A., Nascimento A.M.A., Franco B.D.G.d.M. (2022). Investigating the influence of Food Safety Management Systems (FSMS) on microbial diversity of Canastra cheeses and their processing environments. Food Microbiol..

[72] Li X., Wang H., Abdelrahman H., Kelly A., Roy L., Wang L. (2024). Profiling and source tracking of the microbial populations and resistome present in fish products. Int. J. Food Microbiol..

[73] Hospodsky D., Pickering A.J., Julian T.R., Miller D., Gorthala S., Boehm A.B., Peccia J. (2014). Hand bacterial communities vary across two different human populations. Microbiology.

[74] Edmonds-Wilson S.L., Nurinova N.I., Zapka C.A., Fierer N., Wilson M. (2015). Review of human hand microbiome research. J. Dermatol. Sci..

[75] Goetsch A.G., Ufearo D., Keiser G., Heiss C., Azadi P., Hershey D.M. (2024). An exopolysaccharide pathway from a freshwater Sphingomonas isolate. J. Bacteriol..

[76] Cherifi T., Arsenault J., Quessy S., Fravalo P. (2022). Co-Occurrence of L. monocytogenes with Other Bacterial Genera and Bacterial Diversity on Cleaned Conveyor Surfaces in a Swine Slaughterhouse. Microorganisms.

[77] Busse H.J., Hauser E., Kämpfer P. (2005). Description of two novel species, *Sphingomonas abaci* sp. nov. and *Sphingomonas panni* sp. nov. Int. J. Syst. Evol. Microbiol..

[78] Lin S.Y., Shen F.-T., Lai W.-A., Zhu Z.-L., Chen W.-M., Chou J.-H., Lin Z.-Y., Young C.-C. (2012). Sphingomonas formosensis sp. nov., a polycyclic aromatic hydrocarbon-degrading bacterium isolated from agricultural soil. Int. J. Syst. Evol. Microbiol..

[79] Maillet A., Bouju-Albert A., Roblin S., Vaissié P., Leuillet S., Dousset X., Jaffrès E., Combrisson J., Prévost H. (2021). Impact of DNA extraction and sampling methods on bacterial communities monitored by 16S rDNA metabarcoding in cold-smoked salmon and processing plant surfaces. Food Microbiol..

[80] Gaillac A., Briandet R., Delahaye E., Deschamps J., Vigneau E., Courcoux P., Jaffrès E., Prévost H. (2022). Exploring the Diversity of Biofilm Formation by the Food Spoiler *Brochothrix thermosphacta*. Microorganisms.

[81] de Paiva Anciens Ramos G.L., Vigoder H.C., dos Santos Nascimento J. (2021). *Kocuria* spp. in Foods: Biotechnological Uses and Risks for Food Safety. Appl. Food Biotechnol..

[82] Kloos W.E., Ballard D.N., George C.G., Webster J.A., Hubner R.J., Ludwig W., Schleifer K.H., Fiedler F., Schubert K. (1998). Delimiting the genus *Staphylococcus* through description of *Macrococcus caseolyticus* gen. nov., comb. nov. and *Macrococcus equipercicus* sp. nov., *Macrococcus bovicus* sp. nov. and *Macrococcus carouselicus* sp. nov. Int. J. Syst. Bacteriol..

[83] Götz F., Bannerman T., Schleifer K.-H. (2006). The Genera *Staphylococcus* and *Macrococcus*. The Prokaryotes.

[84] Mazhar S., Hill C., McAuliffe O. (2018). The Genus *Macrococcus*: An Insight I#into Its Biology, Evolution, and Relationship with *Staphylococcus*. Adv. Appl. Microbiol..

[85] Ramos G.L.P.A., Vigoder H.C., Nascimento J.S. (2021). Technological Applications of *Macrococcus caseolyticus* and its Impact on Food Safety. Curr. Microbiol..

[86] Mašlanová I., Wertheimer Z., Sedláček I., Švec P., Indráková A., Kovařovic V., Schumann P., Spröer C., Králová S., Šedo O. (2018). Description and comparative genomics of *Macrococcus caseolyticus* subsp. *hominis* subsp. nov., *Macrococcus goetzii* sp. nov., *Macrococcus epidermidis* sp. nov., and *Macrococcus bohemicus* sp. nov., Novel Macrococci from human clinical material with virulence potential and suspected uptake of foreign DNA by natural transformation. Front. Microbiol..

[87] Schöbitz R., González C., Villarreal K., Horzella M., Nahuelquín Y., Fuentes R. (2014). A biocontroller to eliminate *Listeria monocytogenes* from the food processing environment. Food Control.

[88] Camargo A.C., Todorov S.D., Chihib N.E., Drider D., Nero L.A. (2018). Lactic Acid Bacteria (LAB) and Their Bacteriocins as Alternative Biotechnological Tools to Control *Listeria monocytogenes* Biofilms in Food Processing Facilities. Mol. Biotechnol..

[89] Smith S.A., Newman S.J., Harrison C.E., Loch T.P. (2023). First isolation of *Carnobacterium maltaromaticum* from farmed Rainbow Trout in Virginia. J. Aquat. Anim. Health.

[90] Nan Y., Rodas-Gonzalez A., Stanford K., Nadon C., Yang X., McAllister T., Narváez-Bravo C. (2024). Lactic acid bacteria and spoilage bacteria: Their interactions in *Escherichia coli* O157:H7 biofilms on food contact surfaces and implications for beef contamination. J. Food Saf..

[91] Fagerlund A., Møretrø T., Heir E., Briandet R., Langsrud S. (2017). Cleaning and Disinfection of Biofilms Composed of *Listeria monocytogenes* and Background Microbiota from Meat Processing Surfaces. Appl. Environ. Microbiol..

[92] Pátek M., Grulich M., Nešvera J. (2021). Stress response in *Rhodococcus* strains. Biotechnol. Adv..

[93] Calliauw F., Horemans B., Broekaert K., Michiels C., Heyndrickx M. (2016). Spoilage potential of *Vagococcus salmoninarum* in preservative-free, MAP-stored brown shrimp and differentiation from *Brochothrix thermosphacta* on streptomycin thallous acetate actidione agar. J. Appl. Microbiol..

[94] Rodríguez-López P., Saá-Ibusquiza P., Mosquera-Fernández M., López-Cabo M. (2015). *Listeria monocytogenes*-carrying consortia in food industry. Composition, subtyping and numerical characterisation of mono-species biofilm dynamics on stainless steel. Int. J. Food Microbiol..

[95] Alvarez-Molina A., Cobo-Díaz J.F., Alexa E.A., Crispie F., Prieto M., López M., Cotter P.D., Alvarez-Ordóñez A. (2023). Sequencing-based analysis of the microbiomes of Spanish food processing facilities reveals environment-specific variation in the dominant taxa and antibiotic resistance genes. Food Res. Int..

[96] Shintani H. (2015). Validation Studies for Microbial Contamination and Control of Contaminants. Biocontrol. Sci..

[97] Stiles M.E., Holzapfel W.H. (1997). Lactic acid bacteria of foods and their current taxonomy. Int. J. Food Microbiol..

[98] Fidan H., Esatbeyoglu T., Simat V., Trif M., Tabanelli G., Kostka T., Montanari C., Ibrahim S.A., Özogul F. (2022). Recent developments of lactic acid bacteria and their metabolites on foodborne pathogens and spoilage bacteria: Facts and gaps. Food Biosci..

[99] Pang X., Song X., Chen M., Tian S., Lu Z., Sun J., Li X., Lu Y., Yuk H. (2022). Combating biofilms of foodborne pathogens with bacteriocins by lactic acid bacteria in the food industry. Compr. Rev. Food Sci. Food Saf..

[100] Ghanbari M., Jami M., Domig K.J., Kneifel W. (2013). Seafood biopreservation by lactic acid bacteria—A review. LWT-Food Sci. Technol..

[101] Castellano P., Ibarreche M.P., Massani M.B., Fontana C., Vignolo G.M. (2017). Strategies for Pathogen Biocontrol Using Lactic Acid Bacteria and Their Metabolites: A Focus on Meat Ecosystems and Industrial Environments. Microorganisms.

[102] Hossain M.I., Mizan F.R., Ashrafudoulla, Nahar S., Joo H.-J., Jahid I.K., Park S.H., Kim K.-S., Ha S.-D. (2020). Inhibitory effects of probiotic potential lactic acid bacteria isolated from kimchi against *Listeria monocytogenes* biofilm on lettuce, stainless-steel surfaces, and MBEC^TM^ biofilm device. LWT.

[103] Toushik S.H., Kim K., Ashrafudoulla, Mizan F.R., Roy P.K., Nahar S., Kim Y., Ha S.-D. (2021). Korean kimchi-derived lactic acid bacteria inhibit foodborne pathogenic biofilm growth on seafood and food processing surface materials. Food Control.

[104] Stupar J., Holøymoen I.G., Hoel S., Lerfall J., Jakobsen A.N., Rustad T. (2021). Diversity and Antimicrobial Activity towards *Listeria* spp. and Escherichia coli among Lactic Acid Bacteria Isolated from Ready-to-Eat Seafood. Foods.

[105] Zhang H., Xu J., Chen Q., Wang H., Kong B. (2021). Physiological, Morphological and Antioxidant Responses of Pediococcus pentosaceus R1 and Lactobacillus fermentum R6 Isolated from Harbin Dry Sausages to Oxidative Stress. Foods.

[106] Banerji R., Karkee A., Saroj S.D. (2022). Bacteriocins against Foodborne Pathogens (Review). Appl. Biochem. Microbiol..

[107] Alara J.A., Alara O.R. (2024). Antimicrobial and anti-biofilm potentials of biosurfactants. Industrial Applications of Biosurfactants and Microorganisms: Green Technology Avenues from Lab to Commercialization.

[108] Schmidt R.H., Piotter H.M. (2020). The Hygienic/Sanitary Design of Food and Beverage Processing Equipment. Food Engineering Series.

[109] de Oliveira C.A.F., da Cruz A.G., Tavolaro P., Corassin C.H. (2025). Food Safety: Good Manufacturing Practices, Sanitation Standard Operating Procedures, Hazard Analysis, and Critical Control Point. Antimicrobial Food Packaging.

[110] Quinto E.J., Caro I., Villalobos-Delgado L.H., Mateo J., De-Mateo-silleras B., Redondo-Del-río M.P. (2019). Food Safety through Natural Antimicrobials. Antibiotics.

[111] Nag M., Lahiri D., Dey A., Sarkar T., Pati S., Joshi S., Bunawan H., Mohammed A., Edinur H.A., Ghosh S. (2022). Seafood Discards: A Potent Source of Enzymes and Biomacromolecules with Nutritional and Nutraceutical Significance. Front. Nutr..

[112] Guerrieri E., de Niederhäusern S., Messi P., Sabia C., Iseppi R., Anacarso I., Bondi M. (2009). Use of lactic acid bacteria (LAB) biofilms for the control of *Listeria monocytogenes* in a small-scale model. Food Control.

